# An Atypical Presentation of Molluscum Contagiosum in a Newborn

**DOI:** 10.7759/cureus.63932

**Published:** 2024-07-05

**Authors:** Neha Arora, Ashley Wittmer, Maleka Najmi, Jessica Justus, Sophia Hendrick

**Affiliations:** 1 Medicine, Texas A&M School of Medicine, Bryan, USA; 2 Department of Dermatology, Baylor Scott & White Medical Center, Temple, USA; 3 Department of Pediatrics, Baylor Scott &amp; White McLane Children’s Medical Center, Temple, USA

**Keywords:** pediatric dermatology, molluscum contagiosum neonatal, pox virus, viral infection, molluscum contagiosum

## Abstract

Molluscum contagiosum (MC) is a skin infection caused by a poxvirus that is highly contagious and common among children. When MC does occur in children less than one year old, it is suspected to be a result of vertical transmission through maternal MC infection. In this report, we describe a case of MC on the scalp of a 10-month-old child that started shortly after birth via Cesarean delivery. To our knowledge, this is the first case of MC in a neonate born via Cesarean delivery without evidence of maternal vertical transmission.

## Introduction

Molluscum contagiosum (MC) is a skin infection caused by a poxvirus that leads to dome-shaped, flesh colored, and umbilicated papules [1]. These papules have an incubation period that ranges from two to six months and their course is benign and self-limited in nature [2]. Infection with the MC virus is presumed to occur through direct contact with infected skin, fomites, or auto-inoculation. MC lesions in the genital areas of adults and adolescents are thought to be a result of sexual contact. The prevalence of MC in the general US population is estimated to be 5%, whereas the prevalence in children in the United States is disproportionately higher. At any given time, the point prevalence of MC in children in the United States may fall between 5.1% and 11.5% [3]. Within the pediatric population, children between the ages of one and four are most commonly affected [3,4]. The occurrence of MC congenitally or in neonates is uncommon [5].

Here, we present a case of MC infection on the scalp of a 10-month-old male infant; the infection developed shortly after his birth via Cesarean delivery. The patient’s mother had no evidence of MC infection before, during, or after delivery.

## Case presentation

The patient was a 10-month-old late preterm male who presented to the clinic for evaluation of lesions on his scalp. His parents reported that the lesions appeared a few days after birth; there was no success with the treatment that included mupirocin 2% ointment and hydrocortisone 2.5% ointment. The patient was born at 36 weeks' gestation with a documented history of scalp trauma at birth due to the use of a fetal scalp monitor. His mother had a history of chorioamnionitis that resulted in obstructive labor requiring an urgent cesarean delivery. Nonetheless, forceps and vacuum instruments were not used. There was initially an erosion on the scalp that resolved, but the bumps remained. The patient’s mother denied any history of vaginovulvar lesions, and both parents denied cutaneous manifestations suspicious for MC. The patient did not have any other significant close contacts and did not attend day care.

**Figure 1:**
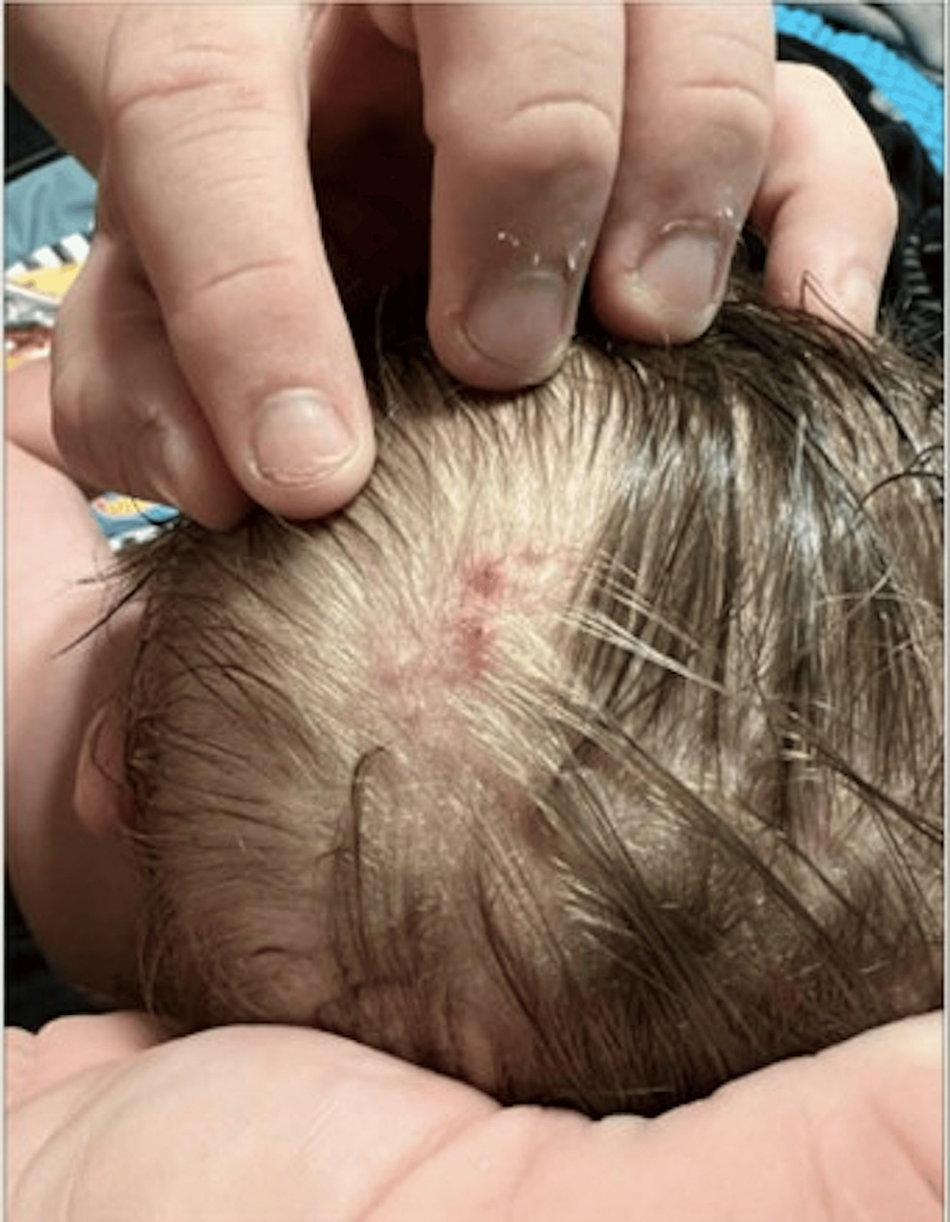
Clustered pink papules, some with central crusting present on the left scalp crown of the 10-month-old patient

**Figure 2:**
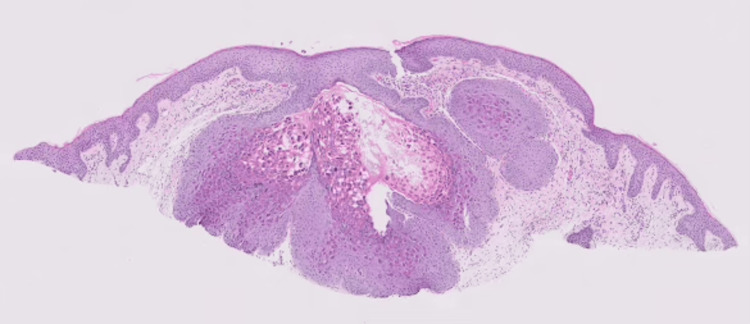
Histopathology revealing epidermal hyperplasia and intracytoplasmic eosinophilic inclusions (molluscum bodies) in the epidermis (hematoxylin and eosin stained)

At the dermatology clinic, a physical examination demonstrated six folliculocentric flesh-colored papules with some overlying crust present on the left crown of the scalp (Figure 1). A shave biopsy was performed and pathology identified the lesions as molluscum contagiosum (Figure 2).

The patient's parents were notified of the biopsy results. They were advised that treatment of these lesions was not necessary, but that it was an option. No further treatment was provided in this case.

## Discussion

MC is a common cutaneous infection most often seen in children, sexually active young adults, and immunocompromised individuals [4]. MC enters the epidermis through breaks in the skin, which is the suspected cause of increased infection in individuals with atopic dermatitis, ichthyosis, or any form of skin barrier compromise [3].

Data on MC in neonates is limited. A case series and literature review published in 2015 reported five cases of neonatal MC, two of which had documented maternal MC lesions [5]. As opposed to the common spread of MC through direct contact, vertical transmission via genital MC during delivery was suspected [5]. In patients without evidence of maternal infection, the possibility of undiagnosed maternal MC is favored heavily [5]. While the remaining three cases of neonatal MC had no evidence of maternal lesions, they were vacuum-assisted vaginal deliveries where the MC lesions were distributed in an annular pattern consistent with placement of the delivery suction instruments [5]. Given the reported link between the spread of MC and the Koebner phenomenon, it is possible that scalp trauma during delivery initiated lesions in neonates without clinically apparent maternal infection [5,6].

The transmission of MC in neonates is thought to occur in the intrapartum period, especially in an infected birth canal following a premature rupture of membranes [7]. Of note, all documented cases of neonatal MC have been vaginal deliveries, regardless of the presence of confirmed maternal MC infection [5,7]. Additional risk factors for vertical transmission include prolonged delivery, primigravida mother, and increased neonatal birth weight or cranial size [5]. While there are treatment options for MC, treatment is not routinely recommended. Similar to our case, many patients do not receive treatment following a diagnosis of MC.

One limitation of this case is that other sources of infection with MC virus such as possible spread via fomites or autoinoculation due to a prior abrasion cannot be completely ruled out. In future cases, disorders that could make a patient more susceptible to the infection such as eczema or T-cell disorders should be further explored.

## Conclusions

Unlike the cases mentioned in the earlier section, our patient did not have maternal MC lesions, instrument-assisted delivery, or vaginal birth. Possibilities for MC transmission in our patient include ascending infection, trauma related to the use of the fetal scalp monitor, or skin-to-skin contact with an unknown source.

Neonatal MC is a rare and underreported diagnosis. Unlike MC in older children, the mechanism of transmission is not fully understood. To our knowledge, this is the first case of neonatal MC in a cesarean delivery without evidence of vertical transmission.
